# Memory and Memory-Like NK Cell Responses to Microbial Pathogens

**DOI:** 10.3389/fcimb.2020.00102

**Published:** 2020-03-25

**Authors:** Marc Brillantes, Aimee M. Beaulieu

**Affiliations:** ^1^Center for Immunity and Inflammation, New Jersey Medical School, Rutgers Biomedical and Health Sciences, Rutgers—The State University of New Jersey, Newark, NJ, United States; ^2^Department of Microbiology, Biochemistry, and Molecular Genetics, New Jersey Medical School, Rutgers Biomedical and Health Sciences, Rutgers—The State University of New Jersey, Newark, NJ, United States; ^3^School of Graduate Studies, Rutgers Biomedical and Health Sciences, Rutgers—The State University of New Jersey, Newark, NJ, United States

**Keywords:** natural killer cells, memory NK cells, memory-like NK cells, adaptive NK cells, infection

## Abstract

NK cells are cytotoxic lymphocytes that provide systemic defense against pathogens and malignancy. Although historically considered cells of the innate immune system, NK cells are now known to be capable of memory or memory-like immune responses in certain settings. Memory NK responses were initially reported over a decade ago in studies involving mouse models of cytomegalovirus infection and delayed-type hypersensitivity reactions to chemical haptens and viral antigens. Since then, a growing body of literature suggests that memory or memory-like NK cell responses may occur in a broader range of immunological settings, including in response to various viral and bacterial infections, and some immunization protocols. Memory-like NK cell responses have also now been reported in humans and non-human primates. Here, we summarize recent studies demonstrating memory or memory-like responses by NK cells in settings of infection and immunization against infectious agents.

## Introduction

Natural killer (NK) cells are cytotoxic lymphocytes specialized for immunological defense against malignant cells and intracellular pathogens. NK cells possess a repertoire of germline-encoded activating and inhibitory receptors that recognize self– and/or microbe–encoded molecules expressed on the surface of host cells (Vivier et al., 2011). Specific receptors include the killer cell immunoglobulin-like receptors (KIRs) in humans, the Ly49 receptors in mice, and natural cytotoxicity receptors (NCRs) and NKG2 receptors in both species. Major histocompatibility complex class I (MHC-I) molecules, which are present on most healthy host cells, are key ligands for inhibitory NK receptors, whereas stress-induced and/or pathogen-encoded molecules upregulated on malignant or infected cells are important ligands for activating NK receptors. NK cells also express Fcγ receptors that engage the Fc portion of antibodies, triggering NK cell activation and antibody-dependent cellular cytotoxicity (ADCC). Overall, NK cell activation is modulated by the balance of signals received through inhibitory and activating receptors. Once activated, NK cells secrete cytolytic molecules, such as perforin and granzymes, to directly lyse target cells. In addition to their cytotoxic functions, activated NK cells are an important source of chemokines and pro-inflammatory cytokines, particularly IFN-γ, which serve to shape and amplify the overall immune response.

NK cells have historically been considered innate lymphocytes, owing to their rapid effector responses even in the absence of prior antigen exposure and their lack of somatically rearranged antigen receptors. NK cells share many developmental and functional attributes with Group 1 “helper” innate lymphoid cells (ILC1s), although their development from distinct progenitor cell populations under homeostatic conditions suggests that they are indeed distinct lineages (Vivier et al., 2018). Notwithstanding the traditional designation of NK cells as innate immune cells, studies over the past decade provide compelling evidence that some NK cell responses exhibit features of adaptive immunity, including clonal-like expansion of antigen-specific effector cells and generation of long-lived memory populations capable of enhanced recall responses. Here, we will use the term “memory” to describe enhanced (or otherwise reprogrammed) responses that are cell-intrinsic, long-lasting, and antigen-specific; “memory-like” will describe responses in which any of the three latter criteria are lacking or unknown.

Antigen-specific memory responses by NK cells were first recognized over a decade ago in independent studies in mice involving murine cytomegalovirus (MCMV) infection (Sun et al., 2009) and delayed-type hypersensitivity reactions to chemical haptens and viral antigens (O'Leary et al., 2006; Paust et al., 2010). Since then, a growing body of literature suggests that memory-like NK cell responses may occur in response to a broader range of viral, bacterial, and possibly even eukaryotic pathogens. Moreover, memory-like NK responses are not limited to experimental mouse models, but have now been described in humans and non-human primates in settings of infection and immunization. Here, we provide an up-to-date overview of studies on adaptive NK cell responses to microbial infections in mice, humans, and non-human primates.

## Memory and Memory-like NK Cell Responses to Viral Infection

### Cytomegalovirus (CMV)

NK cells provide critical host defense against viral pathogens, particularly herpesviruses and papillomaviruses (Orange, 2002). Individuals with deficiencies in functional NK cells are highly susceptible to recurrent, systemic, and even life-threatening herpesvirus infections, and to severe consequences of papillomavirus infections (Biron et al., 1989; Orange, 2002; Etzioni et al., 2005; Notarangelo and Mazzolari, 2006; Mace et al., 2013). NK cells also confer protection against herpesvirus infections in mice. In some mouse strains (e.g., C57BL/6 mice), resistance to the herpesvirus, murine CMV (MCMV), is mediated by a subset of NK cells bearing the NK activating receptor, Ly49H, which recognizes the virus-encoded glycoprotein, m157, expressed on the surface of MCMV-infected cells (Brown et al., 2001; Arase et al., 2002; Smith et al., 2002). MCMV infection triggers the activation and proliferative clonal-like expansion of Ly49H^+^ NK cells. The Ly49H^+^ NK cell response to MCMV is reminiscent of a virus-specific CD8^+^ T cell response, with effector cell expansion peaking at ~day 7 post-infection and a subsequent phase of programmed cell death leading to the removal of most but not all MCMV-experienced Ly49H^+^ NK cells (Sun and Lanier, 2011). Those that remain form a small, long-lived pool of memory cells that can provide superior protection against secondary challenge with MCMV (Sun et al., 2009). MCMV-induced memory NK cells are predominantly Ly6C^hi^DNAM1^lo^CD27^−^CD11b^hi^KLRG1^hi^, and arise from a pool of KLRG1^lo^ cells present within the effector Ly49H^+^ population (Sun et al., 2009; Nabekura et al., 2014; Kamimura and Lanier, 2015). Effector cell expansion and memory cell generation are dependent on the Ly49H receptor and its viral ligand, m157, consistent with a *bona fide* antigen-specific memory response (Sun et al., 2009).

Since the initial description of MCMV-specific NK cell memory over a decade ago, our understanding of the molecular pathways that shape this response has advanced significantly. These advances, which have been comprehensively summarized in several recent reviews (Rapp et al., 2018; Brillantes and Beaulieu, 2019), include findings of positive regulators of memory cell formation, such as IL-12, the costimulatory molecule DNAM1, the transcription factors STAT4, T-bet, Eomes, Runx1, and CBF-β, the microRNA miR-155, and the pro-mitophagy proteins BNIP3/BNIP3L, as well as negative regulators such as the pro-apoptotic molecule, Bim. Effector-to-memory NK cell differentiation is accompanied by transcriptional and epigenetic alterations, such as increased abundance of *Ly6c1* (encoding Ly6C) and *Gzmb1* (encoding Granzyme B) transcripts and enhanced chromatin accessibility at the *Prf1* (encoding Perforin-1) locus, which support the distinct phenotype and functions of memory NK cells (Bezman et al., 2012; Lau et al., 2018).

Analogous to the MCMV-specific memory responses demonstrated in mice, memory-like NK cell populations have also been described in humans with a history of human cytomegalovirus (HCMV) infection. Compared to their HCMV-seronegative counterparts, HCMV-seropositive individuals harbor an expanded population of NK cells expressing the receptor complex comprised of CD94 and NKG2C (Guma et al., 2004). NKG2C^+^ NK cells transferred into patients in settings of hematopoietic stem cell transplantation expand and mount potent IFN-γ responses during HCMV reactivation (Foley et al., 2012a,b). These expanded NKG2C^+^ cells have a unique surface phenotype, with preferential expression of the maturation marker, CD57, and of inhibitory immunoglobulin-like transcript 2 (ILT2) and KIRs, but reduced expression of the NCRs, NKp30 and NKp46, the intracellular signaling proteins, SYK and EAT-2, and the transcription factor, PLZF (Guma et al., 2004; Lopez-Verges et al., 2011; Schlums et al., 2015). HCMV-seropositive individuals also harbor more FcεRIγ^−^ NK cells—many but not all of which are NKG2C^+^–that exhibit enhanced ADCC functionality and IFN-γ production following exposure to antibody-coated target cells (Hwang et al., 2012; Zhang et al., 2013; Lee et al., 2015). The unique phenotype and function of these HCMV-expanded NK cells are mirrored by epigenetic modifications at regulatory regions for the genes encoding FcεRIγ, IFN-γ, EAT-2, and PLZF (Luetke-Eversloh et al., 2014; Lee et al., 2015; Schlums et al., 2015). For example, loss of silencing DNA methylation marks at a conserved non-coding sequence (CNS) upstream of the *IFNG* promoter correlates with enhanced IFN-γ production by activated memory-like NK cell populations (Luetke-Eversloh et al., 2014). Conversely, increased methylation at the FcεRIγ and EAT-2 gene loci correlates with reduced expression of these proteins in memory-like cells (Schlums et al., 2015). These findings suggest that epigenetic reprogramming is an important mechanism underlying the altered functionality of memory-like NK cell populations.

Although the specific NK receptor-ligand interaction(s) that drive expansion of NKG2C^+^ NK cells during HCMV infection remain incompletely understood, the response is thought to be HCMV-specific. In humans, acute HCMV infection or reactivation is associated with the selective expansion or re-expansion of NKG2C^+^ NK cells (Lopez-Verges et al., 2011; Foley et al., 2012a,b). Likewise, CMV infection drives the selective expansion of NKG2C^+^ NK cells in rhesus macaques (Ram et al., 2018). Co-culture of human peripheral blood lymphocytes with HCMV-infected fibroblasts *in vitro* has been reported to drive the selective expansion of NKG2C^+^ NK cells in some (Guma et al., 2006), although not all studies (Newhook et al., 2017).

Recent work demonstrated that NKG2C^+^ NK cells are responsive to the HCMV-derived peptide, UL40, in complex with the non-classical MHC-I molecule, HLA-E, a known NKG2C ligand (Hammer et al., 2018). Similar to their response to HCMV infection *in vivo*, NKG2C^+^ NK cells stimulated with UL40-HLA-E *in vitro* proliferated, downregulated expression of FcεRIγ, and lost DNA methyl marks at the *IFNG* regulatory region CNS1. These effects were highly sensitive to the UL40 peptide sequence, as minor HCMV strain-specific differences in the sequence altered both the magnitude and quality of the response (Hammer et al., 2018).

Altogether, the studies described above suggest that HCMV-specific memory NK cells exist within the NKG2C^+^ NK cell compartment in HCMV-seropositive individuals. However, the role of the NKG2C receptor itself in formation of memory-like NK cells during HCMV infection is not completely clear. In one study, HCMV-exposed *NKG2C*^−/−^individuals were reported to have fewer CD94^+^ NK cells and, in individuals <10 years old, fewer CD57^+^ NK cells overall. In the younger cohort (<10 years old), anti-HCMV antibody titers were also higher, possibly reflecting poorly controlled HCMV infection (Goodier et al., 2014). In contrast, another study reported that HCMV-exposed *NKG2C*^−/−^ individuals maintain an expanded population of NK cells that, although missing NKG2C expression, retained other memory-associated phenotypic and functional characteristics, including enhanced IFN-γ secretion and a demethylated *IFNG* CNS1 region (Liu et al., 2016). Moreover, *NKG2C*^−/−^ NK cells transferred in the context of clinical transplantation therapies were shown to rapidly expand and mature in response to HCMV infection (Della Chiesa et al., 2014). Thus, while the specific role of NKG2C remains to be fully clarified, it is possible that receptors other than, or in addition to, NKG2C support the differentiation of memory-like NK cells during HCMV infection.

Additional evidence that the NKG2C^+^ NK cell response in HCMV-infected individuals is pathogen-specific comes from studies showing that NKG2C^+^ NK cells do not expand during other herpesvirus infections. For example, neither Epstein-Barr virus (EBV) nor herpes simplex virus (HSV)-2 infections are associated with selective expansion of NKG2C^+^ NK cells (Bjorkstrom et al., 2011b; Hendricks et al., 2014). Furthermore, although expansion of NKG2C^+^ NK cells has been described in people infected with hepatitis B virus, hepatitis C virus, chikungunya virus, and hantavirus, this phenomenon appears to be largely restricted to HCMV-seropositive individuals (Brunetta et al., 2010; Bjorkstrom et al., 2011a; Petitdemange et al., 2011; Beziat et al., 2012). Expansion of NKG2C^+^ NK cells in these settings may reflect antibody-driven proliferation of the FcεRIγ^−^ population that is abundant in HCMV-seropositive individuals, consistent with studies showing that FcεRIγ^−^ NK cells expand *in vitro* following exposure to target cells infected with HCMV, HSV-1, or influenza, but only when virus-specific antibodies are also present (Lee et al., 2015).

### Epstein-Barr Virus (EBV)

EBV is a gammaherpesvirus that latently infects most adults worldwide. EBV typically gains entry through the tonsils and establishes long-term residence in B cells, where it is associated with various B cell malignancies, including Burkitt's lymphoma, Hodgkin's disease, and post-transplant lymphoproliferative disorder (PTLD) (Gru et al., 2015). NK cells are thought to play an important role in controlling EBV-induced B cell transformation (Strowig et al., 2008). Human tonsillar NK cells with a NKG2A^+^CD94^+^CD56^bright^ phenotype were shown to restrict the transformation of autologous B cells exposed to EBV *in vitro*, an activity that was dependent on both IFN-γ and the NCR family activating receptor, NKp44 (Strowig et al., 2008; Lunemann et al., 2013; Jud et al., 2017). In pediatric patients with acute symptomatic EBV infection (also known as infectious mononucleosis), NKG2A^+^CD56^dim^KIR^−^ NK cells were shown to undergo selective expansion and persist in peripheral blood at elevated frequencies for many months (Azzi et al., 2014). They also degranulated with greater frequency than their NKG2A^−^CD56^dim^KIR^+^ and CD56^bright^ counterparts following exposure to B cells infected with actively replicating (although not latent) EBV (Azzi et al., 2014). Similarly, in humanized mice, NKG2A^+^ NK cells have been shown to persist at elevated frequencies in the blood for many weeks after EBV infection (Chijioke et al., 2013). Another group reported that human NKG2A^+^ NK cells were also more responsive to lymphoblastoid cell lines latently infected with EBV (Hatton et al., 2016). Collectively, these findings raise the possibility that the NKG2A^+^ compartment may harbor EBV-specific memory-like NK cells in individuals with a history of EBV infection.

### Herpes Simplex Virus (HSV)

Studies in mice suggest that infection with the alphaherpesvirus, HSV-2, may also elicit protective memory-like NK responses. Prior infection of *Rag1*^−/−^ mice with an attenuated strain of HSV-2 was shown to confer resistance to a later lethal dose of HSV-2, notwithstanding the absence of adaptive T and B cells in these animals. NK cells were required for this resistance, as NK cell depletion during the secondary challenge neutralized the protective effect of prior HSV-2 exposure (Abdul-Careem et al., 2012). NK cells from HSV-2-primed mice also exhibited enhanced functionality *in vitro*, producing more IFN-γ than NK cells from naïve mice when exposed to lysate from HSV-2-infected Vero cells, but not lysate from uninfected control cells (Abdul-Careem et al., 2012). Nevertheless, this enhanced functionality was relatively short-lived (~30 days), in contrast to the months-long memory responses elicited by MCMV infection (Sun et al., 2009; Abdul-Careem et al., 2012).

### Varicella Zoster Virus (VZV)

VZV, another common alphaherpesvirus and the causative agent of chickenpox and shingles, was recently suggested to promote memory-like NK responses in humans. Individuals with a history of chickenpox during childhood were shown to mount robust delayed-type hypersensitivity reactions to intradermally injected VZV antigens (Nikzad et al., 2019). This response involved the selective recruitment of actively degranulating NK cells to the VZV antigen injection site, but not to saline-injected control sites, although the contribution of T and B cells to these responses could not be excluded by the study design. The majority of the VZV-recruited NK cells were CD56^hi^ and more frequently expressed markers associated with tissue-residency, including CXCR6, NKG2D, CD69, and CD62L (Nikzad et al., 2019).

### HIV/SIV

The lentivirus, human immunodeficiency virus (HIV), causes acquired immunodeficiency syndrome (AIDS) in humans. Early studies in mice were the first to demonstrate that immunization with HIV antigens could elicit HIV-specific memory NK cell responses (Paust et al., 2010). In these studies, *Rag1*^−/−^ mice were immunized with viral-like particles (VLPs) containing HIV antigens. Transfer of liver, but not splenic, NK cells from immunized donors into naive *Rag2*^−/−^*Il2rg2*^−/−^ recipients resulted in NK-mediated recall responses that were antigen-specific, occurring only in recipients rechallenged with HIV antigens but not antigens from other viruses such as influenza (Paust et al., 2010). More recently, vaccination of BLT (human bone marrow, liver, and thymus) humanized mice with HIV antigens was shown to elicit human NK-mediated recall responses that were both vaccination-dependent and antigen-specific (Nikzad et al., 2019).

Consistent with findings in mouse studies, HIV-specific memory NK cell responses have also been demonstrated in rhesus macaques infected with or vaccinated against simian immunodeficiency virus (SIV) and simian HIV (SHIV) (Reeves et al., 2015). NK cells from infected or vaccinated, but not naive, macaques were shown to lyse target cells pulsed with the HIV Gag or Env antigens. Lysis was antigen-specific, as the NK cells did not kill target cells pulsed with mismatched antigens. Notably, HIV-specific recall responses persisted for up to 5 years after vaccination, underscoring the longevity of these memory NK cell responses in macaques (Reeves et al., 2015). NK killing of antigen-pulsed targets was dependent, at least in part, on functional signaling through the NKG2A and NKG2C receptors, although whether these receptors mediate direct or indirect recognition of HIV antigens remains unclear (Reeves et al., 2015). Intriguingly, studies involving human NK cells indicate that the human KIR, KIR2DL1, can recognize HIV peptides on infected cells in an HLA-dependent manner (Alter et al., 2011). Moreover, KIR3DS1^+^ and KIR3DL1^+^ NK cells have been shown to selectively expand during acute HIV infection in individuals that express HLA-B Bw4801(Alter et al., 2009). Collectively, these findings suggest that investigation of HIV-specific memory NK responses in humans will be an important focus for future studies.

### Vaccinia Virus

Memory-like NK cell responses to vaccinia virus, a member of the Poxviridae family, have been suggested in studies involving mice and macaques. Adoptively transferred CD90^+^ liver NK cells from mice previously infected with vaccinia were more effective than NK cells from naïve donors at protecting *Rag1*^−/−^ recipients against lethal vaccinia challenge (Gillard et al., 2011). A complementary study in macaques examined NK cell responses elicited by prime-boost immunization protocols involving the modified vaccinia virus Ankara strain. NK cells from “boosted” macaques had higher expression of molecules associated with cytotoxicity, homing, adhesion, and maturity than NK cells from macaques that received the priming challenge only. Importantly, these changes were long-lived, persisting for months after the initial priming event, consistent with a possible memory-like phenotype (Palgen et al., 2019).

### Influenza Virus

Influenza vaccination in humans was shown to increase the frequency of circulating CD56^dim^ NK cells for >1 month post-vaccination (Dou et al., 2015). These CD56^dim^ NK cells had lower surface expression of NKp46 than their CD56^bright^ counterparts and produced more IFN-γ when restimulated *in vitro* with inactivated influenza (Dou et al., 2015). Enhanced IFN-γ production was dependent on functional NKp46 signaling at the time of restimulation, possibly reflecting a role for NKp46 in recognition of influenza antigens as previously suggested (Mandelboim et al., 2001; Dou et al., 2015). Similar observations of enhanced effector responses by peripheral blood NK cells after influenza vaccination were also reported by a separate group (Goodier et al., 2016). In this study, NK cells evaluated up to 3 months after vaccination produced more IFN-γ and degranulated better when restimulated *in vitro* with inactivated influenza and low-dose IL-12 and IL-18. This enhanced functionality was partially dependent on Type I interferon signaling. The latter observation led to the suggestion that Type I interferons elicited by vaccination might alter the activation threshold of NK cells in immunized individuals (Goodier et al., 2016), consistent with prior findings that exposure to pro-inflammatory cytokines, such as IL-12, IL-18, and IL-15, can program long-lasting, albeit antigen-independent, memory-like features in NK cells (Cooper et al., 2009; Romee et al., 2012).

Analogous to findings in humans, mice immunized with sublethal doses of influenza virus were shown to harbor a population of CD49a^+^DX5^−^ NK cells that expressed high levels of the memory-associated markers, Ly6C and KLRG1 (Li et al., 2017). Adoptively transferred liver, but not lung, CD49a^+^DX5^−^ NK cells from influenza-vaccinated mice protected naive mice against a secondary challenge with lethal doses of virus (Li et al., 2017). Similarly, Rag1-deficient mice immunized with VLPs containing influenza antigens were shown to harbor liver-resident memory NK cells capable of protective, virus-specific recall responses (Paust et al., 2010).

## Memory-like NK Cell Responses to Bacterial Pathogens

### *Mycobacteria* Species

Recent studies in mice and humans indicate that NK cells may mediate memory-like responses to *Mycobacterium tuberculosis* (Mtb), the causative agent of tuberculosis (TB). Human NK cells expressing the T cell memory marker, CD45RO, were shown to accumulate in the pleural fluid of TB patients. Compared to CD45RO^−^ NK cells, these CD45RO^+^ NK cells produced more IFN-γ and were more cytotoxic against tumor cells when re-stimulated with IL-12 *in vitro* (Fu et al., 2011). They also produced more IL-22 and IFN-γ when co-cultured with autologous monocytes infected with Bacillus Calmette-Guerin (BCG), a live-attenuated mycobacterium routinely used to vaccinate humans against TB (Fu et al., 2016). Indeed, revaccination of individuals with BCG has been shown to boost the frequency of BCG-reactive NK cells in the peripheral blood for at least a year after revaccination (Suliman et al., 2016).

Similarly, in mice, BCG inoculation expanded a subset of CD27^+^KLRG1^+^ NK cells that underwent selective re-expansion in response to a subsequent challenge with Mtb. Transfer of CD27^+^ NK cells from BCG-primed, but not mock-immunized, mice into naïve animals conferred partial protection against Mtb infection, reducing overall bacterial burdens in the lung. This protection was specific to the CD27^+^ compartment—transferred CD27^−^ NK cells from BCG-primed donors failed to protect recipients from Mtb—and correlated with the enhanced proliferative and IFN-γ responses by transferred CD27^+^ NK cells during Mtb infection. Mechanistically, generation of memory-like NK cells following BCG inoculation required the presence of T cell-derived IL-21 at the time of primary BCG infection (Venkatasubramanian et al., 2017).

In addition to, or perhaps instead of, mycobacterial-specific memory responses, BCG immunization may also confer antigen-independent memory-like features in NK cells, possibly through reprogramming mechanisms involving pro-inflammatory cytokine stimulation, as described in other settings (Cooper et al., 2009; Romee et al., 2012). Indeed, NK cells from BCG-immunized patients were shown to mount enhanced effector responses when restimulated not only with mycobacterial, but also with fungal antigens *in vitro* (Kleinnijenhuis et al., 2014). Additionally, BCG-vaccinated mice were shown to be more resistant than naïve animals to heterologous challenge with the fungal pathogen, *Candida albicans*. Heterologous protection was maintained in vaccinated SCID mice, which lack T and B cells but possess functional NK cells, but was lost in mice lacking all lymphocytes, suggesting that memory-like NK cells contributed to protection in these studies (Kleinnijenhuis et al., 2014).

### *Ehrlichia* Species

Memory-like NK cell responses were recently described in mice infected with the intracellular bacterial pathogen, *Ehrlichia muris*. Prior work had established that mice infected with a non-lethal dose of *E. muris* were resistant to a later challenge with the more virulent *Ehrlichia* strain, *Ixodes ovatus Ehrlichia* (IOE) (Thirumalapura et al., 2008, 2009). A follow-up study demonstrated that NK cells were critical for this resistance, as *E. muris*-primed mice depleted of NK cells prior to secondary IOE challenge rapidly succumbed to infection (Habib et al., 2016). Moreover, transferred NK cells from *E. muris*-primed donor mice, but not NK cells from naïve or IOE-primed donors, were capable of protecting *Rag2*^−/−^*Il2rg2*^−/−^ recipients against a high dose *E. muris* challenge. Protection was maintained even in *Rag2*^−/−^*Il2rg2*^−/−^ recipients co-treated with anti-CD4 antibodies, ruling out a protective role for contaminating CD4^+^ T cells that may have been co-transferred with the NK cells (Habib et al., 2016).

## Memory-like NK Cell Responses to Eukaryotic Pathogens

*Plasmodium falciparum* is a unicellular protozoan parasite that causes malaria in humans. At specific stages of its life cycle, *P. falciparum* infects red blood cells (RBCs), which display parasite-derived proteins on their surface that facilitate vascular adhesion and sequestration. Recent studies identified a unique population of FcεRIγ^−^ NK cells that mediates killing of *P. falciparum*-infected RBCs via ADCC when antibodies that bind *P. falciparum* antigens on the RBC surface are also present (Arora et al., 2018; Hart et al., 2019). These FcεRIγ^−^ NK cells were found to be expanded in malaria-exposed individuals, and their relative abundance correlated with reduced parasitemia and resistance to clinical symptoms of malaria (Arora et al., 2018; Hart et al., 2019). Similar to the memory-like NKG2C^+^ population that expands during CMV infection, the FcεRIγ^−^ population in malaria-exposed subjects largely lacked PLZF expression and exhibited heightened ADCC functionality. However, these FcεRIγ^−^ NK cells were not uniformly NKG2C^+^, and their frequency was similar in both HCMV-seropositive and –seronegative subjects, suggesting they are at least partially distinct from CMV-expanded adaptive NK cells (Hart et al., 2019). Whether the FcεRIγ^−^ population contains genuine *Plasmodium*-specific memory cells, or even non-specific cytokine-induced memory-like cells, remains to be determined.

Of note, evidence of adaptive NK cell responses was found to be conspicuously absent in mice infected with a related apicomplexan parasite, *Toxoplasma gondii*. Although NK cells were important for protection against secondary *T. gondii* challenge, this role was notably independent of any cell-intrinsic differences in functionality. NK cells from mice previously infected with *T. gondii* were comparable to those from naïve mice with respect to longevity and their failure to protect *Rag2*^−/−^*Il2rg*^−/−^ mice from *T. gondii* challenge (Ivanova et al., 2019).

## Memory NK Cells as Vaccine Targets

Current vaccination protocols largely target B and T cells, with the general goal of generating high titers of neutralizing antibodies. However, vaccine strategies that target memory NK responses may be useful in boosting protection, especially against microbes that evade control by neutralizing antibodies. The feasibility of harnessing NK cells in this manner is supported by findings of antigen-specific memory NK responses against diverse classes of antigens, ranging from distinct viral proteins to small chemical haptens (Geary and Sun, 2017). In addition, the enhanced ADCC functionality of some memory-like NK populations, e.g., the FcεRIγ^−^ populations observed in CMV-exposed individuals (discussed in CMV section above), suggests a possible role for adaptive NK cells in boosting the efficacy of vaccine-induced antibodies.

Notwithstanding the potential utility of adaptive NK cells in providing long-lasting immunity, the development of vaccines that elicit pathogen-specific NK responses will be aided by a better understanding of how NK cells gain specificity for diverse antigens in light of their fixed receptor repertoire. To date, little is known about the specific microbial antigen and cognate NK receptor pairs that support NK memory. A notable exception is the recognition of m157 by Ly49H. The gene encoding Ly49H appears to have arisen out of a DNA recombination event involving the inhibitory receptor, Ly49I, which binds MHC class I (Brown and Scalzo, 2008). Given that m157 is a structural mimic of MHC class I (Adams et al., 2007), it may have originally evolved to suppress NK cell function through engagement of Ly49I. Indeed, the activating effects of m157 are limited to mouse strains that evolved to express Ly49H rather than Ly49I (Arase et al., 2002; Smith et al., 2002). Thus, the role of m157 and Ly49H interactions in memory NK responses likely reflects a specific process of co-evolution between MCMV and its mouse host, rather than a general mechanism by which NK cells acquire specificity to diverse antigens. Nevertheless, memory responses against another MCMV-encoded glycoprotein, m12 were recently described for ILC1s (Weizman et al., 2019) and other Ly49 receptors have been shown to recognize MCMV antigens, e.g. recognition of MCMV m04 in the context of H2-D^k^ in MA/MyJ mice (Kielczewska et al., 2009). Thus, the Ly49H memory NK response to m157 is not likely an isolated phenomenon. Whether and how NKG2 receptors contribute to antigen-specific NK responses to HIV antigens in macaques; why NKG2C^+^ and NKG2A^+^ populations are associated with memory-like responses to HCMV and EBV in humans, respectively; and whether the KIR3DS1^+^ and KIR3DL1^+^ NK cells that expand during HIV infection in humans become memory cells are important and open questions in the field. Until we better understand the specific NK receptors and cognate antigens involved in NK memory, vaccination with inactivated or attenuated pathogens may remain the most viable option to maximize the likelihood of generating pathogen-specific responses.

Another important consideration in developing vaccines to target NK cells is related to the finding that unique tissue-resident or tissue-specific populations can contribute to adaptive NK responses (Paust et al., 2010; Nikzad et al., 2019). Understanding how to target these populations might be beneficial in generating protective immunity against pathogens that gain entry through or colonize specific tissues. Toward this end, humanized mouse models might be particularly useful in the experimental interrogation of human tissue-resident NK cells not readily obtained from blood.

## NK Cell Memory: A T Cell Perspective

NK cells share many functional properties with cytotoxic CD8^+^ T cells, and recent studies have highlighted notable similarities in memory cell differentiation as well. As described above, mouse Ly49H^+^ NK cells activated during MCMV infection exhibit distinct activation, expansion, contraction, and memory phases that are similar to classical antiviral CD8^+^ T cell responses. Notably, the signals and molecular pathways that regulate these specific phases also share similarities. For example, the “three-signal” model of T cell activation—TCR activation via antigen (signal 1), co-stimulation (signal 2), and inflammation (signal 3)—has clear parallels in NK cell activation during MCMV infection, with Ly49H:m157 engagement providing signal 1, costimulatory receptors such as DNAM1 providing signal 2, and pro-inflammatory cytokines such as IL-12 providing signal 3. With respect to Signal 1, recent studies suggest that its strength and/or duration not only impacts T cell memory, but also NK cell memory responses (Snook et al., 2018; Adams et al., 2019; Grassmann et al., 2019; Li et al., 2019). Specifically, high-avidity Ly49H^hi^ NK cells were shown to be more proliferative and to preferentially form memory cells following MCMV infection, whereas low-avidity Ly49H^lo^ cells became the principal IFN-γ producers (Adams et al., 2019; Grassmann et al., 2019). Avidity-based memory cell programming may provide a mechanism to ensure that the memory pool is comprised of cells with increased specificity or binding to viral ligand. Other common molecular regulators of T and NK memory responses include the cytokine IL-15 and Bcl-2 family proteins, such as pro-apoptotic Bim and anti-apoptotic Bcl-2 and Mcl-2, which act in balance to ensure proper effector cell apoptosis and memory cell survival during the contraction phase (Grayson et al., 2000; Min-Oo et al., 2014).

Intriguingly, differential expression of the activating receptor, KLRG1, by effector T and NK cells has been shown to distinguish cells with effector vs. memory cell potential at early stages of a viral infection. Among activated T cells, high KLRG1 expression marks short-lived effector cells (SLECs) and low KLRG1 expression marks memory precursor effector cells (MPECs). Similarly, for Ly49H^+^ NK cells activated during MCMV infection, KLRG1^+^ cells appear to reflect terminally differentiated effector cells, whereas KLRG1^low/−^ cells may preferentially seed the memory compartment, analogous to T cell SLECs and MPECs (Kamimura and Lanier, 2015). For T cells, MPECs give rise to central memory (T_cm_) or effector memory (T_em_) T cells, which preferentially home to lymphoid organs or remain in circulation, respectively. Whether a similar “division of labor” occurs among Ly49H^+^ memory NK cells remains unknown, although recently developed single-cell genomics and lineage-tracing tools may prove useful in addressing this question.

## Concluding Remarks

**Figure 1:**
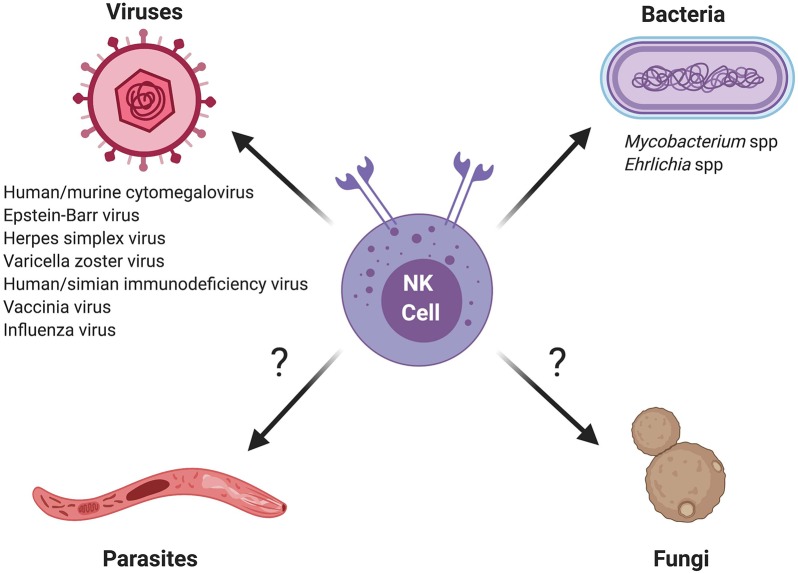
Schematic of memory and memory-like NK cell responses against different classes of microbial pathogens. Question marks indicate eukaryotic pathogens for which memory or memory-like responses by NK cells require further investigation.

In summary, a growing body of evidence suggests that NK cells have the capacity to mount memory or memory-like responses to a diverse range of viral and bacterial pathogens, and possibly even eukaryotic pathogens such as *Plasmodium falciparum* (Table 1 and Figure 1). It will be interesting to see whether future studies uncover memory NK responses against other eukaryotic organisms, and in particular the various fungal pathogens controlled by NK-mediated host defenses (reviewed in Ogbomo and Mody, 2016; Mody et al., 2019).

**Table 1:** Memory and memory-like NK cell populations in mice, humans, and non-human primates.

**Pathogen/Microbial Antigen**	**Species**	**Memory cell markers**	**Select references**
MCMV	Mouse	Ly49H^+^Ly6C^hi^KLRG1^hi^ CD43^hi^DNAM1^−/lo^CD27^lo^ CD11b^+^	Sun et al., 2009; Nabekura et al., 2014; Kamimura and Lanier, 2015
HCMV	Human	NKG2C^+^CD94^+^CD57^+^ILT2^+^ NKp46^lo^NKp30^lo^SYK^lo^EAT-2^lo^ PLZF^lo^FcεRIγ^lo^	Guma et al., 2004, 2006; Lopez-Verges et al., 2011; Beziat et al., 2012; Foley et al., 2012a,b; Zhang et al., 2013; Lee et al., 2015; Schlums et al., 2015
EBV	Human	NKG2A^+^KIR^−^CD56^dim^	Chijioke et al., 2013; Azzi et al., 2014; Hatton et al., 2016
HSV-2	Mouse		Abdul-Careem et al., 2012
VZV	Human	CD56^hi^CXCR6^+^	Nikzad et al., 2019
HIV^a^	Mouse/humanized mouse	CXCR6^+^	Paust et al., 2010; Nikzad et al., 2019
SIV/SHIV	Rhesus macaque		Reeves et al., 2015
Vaccinia virus	Mouse	CD90^+^	Gillard et al., 2011
Influenza virus	Human	CD56^dim^NKp46^lo^	Dou et al., 2015
Influenza virus	Mouse	CD49a^+^CD49b^−^KLRG1^hi^Ly6C^hi^CD62L^lo^	Paust et al., 2010; Li et al., 2017
*Mycobacterium* spp.	Human	CD45RO^+^CD27^+^	Fu et al., 2011, 2016; Venkatasubramanian et al., 2017
*Mycobacterium* spp.	Mouse	CD27^+^	Venkatasubramanian et al., 2017
*Ehrlichia* spp.	Mouse		Habib et al., 2016
*Plasmodium falciparum (?)*	Human	PLZF^lo^FcεRIγ^lo^	Hart et al., 2019

a *Mice were immunized with HIV antigens Gag and/or Env*.

In some experimental models, memory NK responses are clearly pathogen-specific. In others, inflammatory signals that arise during infection or immunization protocol may reprogram NK cells, such that subsequent responses to both specific and non-specific stimuli are enhanced or altered. Indeed, exposure to pro-inflammatory cytokines is known to imprint long-lived memory-like features in both human and mouse NK cells (Cooper et al., 2009; Romee et al., 2012). The collective body of literature suggests that individuals likely harbor multiple, distinct pools of memory of memory-like NK populations, which may act independently or cooperatively to confer long-term immunity against different pathogens. Future studies are needed to understand the common and unique features of these various memory-like populations, as well as the molecular and epigenetic pathways that program them. Ultimately, a better understanding of NK cell memory could inform clinical efforts to harness their capabilities in vaccination strategies or cellular immunotherapies, particularly in settings where memory responses by T cells and/or B cells are inadequate to prevent infection or malignancy.

## Author Contributions

MB and AB wrote the manuscript and designed the Figure and Table.

### Conflict of Interest

The authors declare that the research was conducted in the absence of any commercial or financial relationships that could be construed as a potential conflict of interest.
